# Ten-week lifestyle changing program reduces several indicators for metabolic syndrome in overweight adults

**DOI:** 10.1186/1758-5996-4-1

**Published:** 2012-01-19

**Authors:** Marita S Mecca, Fernando Moreto, Franz HP Burini, Reinaldo C Dalanesi, Kátia CP McLellan, Roberto C Burini

**Affiliations:** 1 Department of Public Health - Medical School - Sao Paulo State University - UNESP, Brazil

## Abstract

We aim to investigate the effectiveness of a 10-week lifestyle intervention focusing on physical activity and high fiber intake for reducing indicators for metabolic syndrome in overweight-obese individuals. A prospective study of 50 overweight (OW) adults (22 in the general educational group - G1; 28 in the high fiber nutrition group - G2) was performed. Both groups were offered dietary counseling and supervised exercise. Clinical, anthropometric, dietary and plasma biochemical tests were performed at baseline - time 0 (T0) and after 10 weeks - time 1 (T1). Both groups improved their dietary quality, but only G2 presented higher intake of fruit and vegetables (servings/day), higher plasma β-carotene levels and a 24% reduction of MetS incidence. Additionally G2 showed greater reductions in body fat (4%), and waist circumference (7%), obesity class III (2%) and obesity class II (14%) rate. Lifestyle intervention, including a high dietary fiber intake, improved healthy eating index and decreased body fat composition and plasma lipid concentrations leading to MetS incidence reduction.

## Introduction

The metabolic syndrome (MetS) is a complex disorder characterized by abdominal obesity, insulin resistance, hypertension, hypertriglyceridemia and low HDL-c concentration, and inflammation [1]. Four major components identify metabolic syndrome: central obesity, hypertriglyceridemia and low high density lipoprotein cholesterol (HDL-c) concentration, elevated blood pressure and elevated plasma glucose levels [2]. These major components are often associated with decreased insulin sensitivity [3], unhealthy body composition [4], a pro-inflammatory [5], pro-oxidant [6] and prothrombotic [2] states and a low level of cardio-respiratory fitness [7]. Hence sedentary behavior and obesity are strongly associated with an increased risk for developing metabolic syndrome [3,8].

MetS is associated with severe health complications, such as increased risk of type 2 diabetes and atherosclerotic cardiovascular disease [9] and represents a growing public health problem [10]. Development of the MetS is influenced by genetic as well as environmental factors [11,12]. MetS increases the risk of premature death [13-15], therefore, effective and affordable strategies to control the syndrome would benefit the population at risk.

In the western world ~25% of young to middle-aged adults have MetS [16]. There seem to be strong age dependence in the prevalence of metabolic syndrome, but the incidence rises rapidly within adolescents and middle-aged groups and follows the development of obesity in the general population [17].

Lifestyle modification focusing on improving dietary quality and physical activity is the preferred first-line treatment for the management of metabolic syndrome components and comorbidities. However, attempts to modify lifestyle to improve dietary quality and physical fitness are often unsuccessful due to low compliance. Simpler approaches to diet and physical activity have been under investigation [18].

To date, most researchers agree that overweight and obesity are caused by numerous factors; however, a long-term energy imbalance between intake and expenditure appears to be the primary cause. Hence, limiting energy consumption would be a key objective when weight loss is the goal. Regular physical activity can improve the metabolic profile and the risks of cardiovascular diseases and premature mortality. Physical exercises either aerobic interval training or strength training or even a combination of both have beneficial effects on physiological abnormalities associated with MetS [19]. Over the years, much attention has been devoted to prescribing the optimal amount of energy intake to achieve a healthy body weight. Some researchers have looked even further and investigated the possibility that consuming certain dietary factors may aid in weight regulation. Fiber intake is a dietary factor that has received substantial attention by several scientists [20-22].

The present study examined the effectiveness of a 10-week lifestyle intervention focusing on physical activity and high fiber intake for reducing indicators for metabolic syndrome in overweight-obese individuals.

## Subjects and Methods

### Study population

The Lifestyle Changing Program (LCP) that was offered to patients with non-communicable chronic diseases in Botucatu city (São Paulo, Brazil) consisted of supervised exercise and nutritional counseling and has been described elsewhere [23]. The studied subjects were part of subgroup (convenience sample) of participants clinically screened for the lifestyle change program "*Mexa-se Pró-Saúde *[Move for Health]", in the year of 2006.

A total of 50 overweight-obese individuals (11 men and 39 women), (Body mass index = 33.0 ± 5.1 kg/m^2^) and with a mean age of 50.2 ± 11.8 years old were included in this study. This study was conducted in accordance with the Declaration of Helsinki (1964). Subjects gave their written consent to participate in the study, which was approved by the Medical Ethics Committee of Sao Paulo State University (Comitê de Ética em Pesquisa da Faculdade de Medicina da Universidade Estadual Paulista - UNESP).

### Study Design

The study was longitudinal quasi-experimental with evaluations at baseline - time 0 (T0) and after a 10-week of lifestyle intervention based on physical exercises and dietary counseling - time 1 (T1). Participants of the lifestyle changing program were divided in two groups: general education group (G1) of 22 subjects who were given dietary counseling at baseline and physical activity section three times a week; and high fiber nutrition group (G2) of 28 subjects who decided to follow a high dietary fiber intake (30 g/day) along with weekly dietary counseling and physical activity section three times a week. The enrollment for both groups was voluntary. All participants were invited to attend the physical examination at baseline and after 10 weeks. Biochemical data, anthropometric data, and dietary intake were assessed at baseline and T1.

Dietary counseling was provided by a dietitian that met with all participants at baseline to discuss the dietary intervention for both groups. The dietary intervention for the general education group (G1) consisted of general group discussion about benefits of fruit and vegetable intakes and body weight loss.

The dietary intervention for the high fiber nutrition group (G2) consisted of a nutritional counseling group about fiber-rich foods once a week focused on increasing the intake of fruits and vegetables. Participants were given a target number of daily fiber intake of 30 g [24]. At each group visit with the dietitian participants were given a list and description of whole grain foods to help them identify foods to include in their diet. The weekly group section also discussed ingredients substitution in meals to increase fiber intake through adding fruits and vegetables to the usual recipes and different cooking methods. In addition, participants were encouraged to increase the daily intake of fruit and vegetables, whole grain cereals, legumes, low-fat dairy products, and lean meat, fish or poultry as recommended in the Food Guide for the Brazilian population [25].

### Laboratory analyses

Blood samples were collected by vacuum venous puncture, after a 10 to 12-hour fasting period, and centrifuged to obtain serum and plasma samples which were stored at-80°C until the end of the study. The individuals were previously advised to not perform vigorous physical exercises 24-hours and/or consume alcohol 72-hours prior to blood collection. Plasma triglycerides (TG), total cholesterol (Total-c), high-density lipoprotein cholesterol (HDL-c), uric acid (UA), creatinine (Cr) and gamma-glutamyl transpeptidase (γGT) were assayed by dry-chemistry (Systems Vitros chemistry 950 Xr). Plasma low-density lipoprotein cholesterol (LDL-c) was calculated using the Friedewald equation, β-carotene (βC) and malonildyaldehyde (MDA) were assayed by high-performance liquid chromatography and the high-sensitive C-reactive protein (hS-CRP) by chemoluminescense (Immulite, 2000).

### Body composition

Weight, height and waist circumference (WC) were measured with standardized protocols [26]. Body Mass Index (BMI) and waist circumference were evaluated according to the World Health Organization [27]. Body fat percentage (BF%) was assessed by a bioelectrical impedance device (Biodynamics^®^, model 450, USA).

The percentage of muscle mass (%MM) was obtained using the Janssen *et al*., [27] equation and the muscle mass index (MMI) was calculated as MM (kg)/height^2^. Individuals were classified as sarcopenic if their values were below 10.75 kg/m^2 ^and 6.75 kg/m^2 ^for men and women, respectively [28].

### Dietary intake

Dietary intake data was assessed using a single 24-hour dietary recall at baseline and T1. The diet was documented by a dietitian, and to obtain precise information, the subjects were asked if that was typical day of intake from them, how often they usually ate during the day, what variety of food was consumed, how the food was prepared, what the serving size was, and what the brand of the food/meal was. Total caloric intake was computed using the Brazilian food tables [29-31]. The Healthy Eating Index (HEI) modified for the Brazilian population was used to assess the quality of the participants' diet [32]. Eight food groups and 12 components to measure the variety and quality of food intake were evaluated.

### Physical activity

All participants were submitted to supervised exercise of 80 minutes, including warm up (20 min) walking (40 min)/stretching (20 min), 3 x/wk complemented with 60 min (2 x/wk) of strength (40 min), stretching (10 min) at a gym [33]. Only participants with frequency of 3 x/wk were included in the study.

### Cardio-respiratory fitness

Cardio-respiratory fitness was determined as maximum oxygen consumption (VO_2_max) using an electric treadmill (model QMCTM90) according to the Balke protocol [34]. The respiratory indexes was continually measured by an open circuit ergospirometric system (model QMCTM90 Metabolic Cart, Quinton^®^, Bothel, USA) with the Mix-Chamber method and with constant monitoring of the heart and respiratory rates and blood pressures.

### Metabolic Syndrome

MetS was defined using the criteria of National Cholesterol Education Program (NCEP) Expert Panel on Detection, Evaluation, and treatment of High Blood Cholesterol on Adults (ATP-III) when the individual presented three of more of the following factor: abdominal obesity, elevated plasma glucose (considering fasting serum glucose 100 mg/dL or greater) elevated plasma TG levels, low levels of plasma HDL-c, and high blood pressure [2].

### Blood Pressure

Systolic (SBP) and diastolic (DBP) arterial blood pressure (BP) was evaluated with the individual in the seated position according to the procedures described by the VI Brazilian Guidelines on Arterial Hypertension [35], using properly sized cuffs for arm circumference, considering the width/length proportion of 1:2, and the width of the cuff's rubber bag, which should correspond to 40% of arm circumference, and length, to at least 80%.

### Statistical analysis

Descriptive statistics were performed for the study and continuous variables are presented as means ± standard deviation (SD). Categorical variables are presented as absolute numbers and percentages. Continuous variables were compared by the independent t-test. Samples were tested for normal distribution (Shapiro Wilk) and groups were compared by either Student's t-test or Wilcoxon Mann Whitney test. The results are discussed based on a significance level of 5% (p < 0.05).

## Results

Baseline characteristics of participants did not differ significantly between the groups (Table 1). Baseline values and changes in body composition, dietary intake and laboratory analysis are listed in tables 1 to 3.

**Table 1:** Body composition and clinical measures of control (G1) and high dietary fiber (G2) groups at baseline (T0) and after 10 weeks of intervention (T1).

		T0	T1
Body weight (kg)	G1	85.6 ± 19.1	86.0 ± 19.4
	G2	89.1 ± 19.4	85.4 ± 18.7^a, b^
BMI (kg/m^2^)	G1	32.1 ± 5.3	32.2 ± 5.4
	G2	33.5 ± 5.3	32.1 ± 5.1^a, b^
Body fat (%)	G1	36.7 ± 9.5	36.5 ± 9.3
	G2	38.6 ± 8.8	37.2 ± 8.8^a, b^
WC (cm)	G1	103.2 ± 12.7	103.7 ± 14.0
	G2	106.7 ± 14.4	101.4 ± 14.2^a, b^
Muscle Mass (kg)	G1	25.2 ± 5.9	24.4 ± 6.1
	G2	25.36 ± 6.3	26.8 ± 6.6
SBP (mmHg)	G1	122 ± 16.0	127 ± 16.0
	G2	130 ± 20.5	114 ± 13.5^a, b^
DBP (mmHg)	G1	75.0 ± 4.5	76.5 ± 11.0
	G2	86.2 ± 9.2	77.0 ± 6.5^a, b^
VO_2max_	G1	30.8 ± 8.4	28.6 ± 10.8
	G2	26.1 ± 6.4	25.1 ± 5.7

Data expressed as mean ± SD; BMI: body mass index, WC: waist circumference, SBP: Sistolic Blood Pressure, DBP: Diastolic Blood Pressure, VO_2max_: maximum oxygen consumption;

^a ^statistical significance after intervention (p < 0.05).

^b ^statistical significance between groups (p < 0.05).

Participants in the G2 had significantly greater reductions for waist circumference (-5.0% greater), weight (-4.1% greater), BMI (-4.0% greater) and Body fat mass (-3.6% greater) over the 10-week study period. Both muscle mass and aerobic capacity (VO2max) were kept similarly throughout the experiment. Only G2 experienced a significant decrease in blood pressure systolic (-15.5%) and diastolic (-9.5%) from baseline to post-intervention (Table 1).

The overall pattern of dietary intake improved in both groups but with higher improvements in G2, mainly HEI (31.1%) and intake of fruit and vegetables (279%) (Table 2). The percentage of individuals that reached the recommendation of dietary fiber (30 g/day) after the intervention was 9.0% in the G1 group and 46.4% in the G2 group. The amount of 20 g of dietary fiber per day was reached, after the intervention, for 31.8% of individuals from G1 and 96.4% of individuals from G2.

**Table 2:** Food intake of control (G1) and high dietary fiber (G2) groups at baseline (T0) and after 10 weeks of intervention (T1).

		T0	T1
HEI (points)	G1	83.3 ± 8.3	84.2 ± 8.3
	G2	76.9 ± 15.5	97.2 ± 11.0^a, b^
Legumes (servings/day)	G1	1.82 (0-6.5)	1.11 (0-4)
	G2	1.70 (0-8.5)	1.77 (0-5.5)
Fruits + vegetables (g/day)	G1	240.2 (0-842)	260 (20-918)
	G2	231 (0-937)	539 (127-909)^a, b^
Fruits (servings/day)	G1	1.7 (0-10.5)	2 (0-10)
	G2	1.5 (0-10.5)	4 (0.5-8.5)^a, b^
Vegetables (servings/day)	G1	1 (0-5)	1 (0-5)
	G2	0.5 (0-4)	1.7 (0.5-5.5)^a, b^
Dietary fibers (g/day)	G1	16.1 ± 7.8	17.1 ± 9.4
	G2	15.2 ± 7.0	32.1 ± 8.9^a, b^
Oils (servings/day)	G1	2 (0.5-9.5)	1.5 (0.5-4.5)
	G2	1.9 (0-9.5)	0.75 (0-2.5)^a, b^
Total energy (kcal/kg BW/day)	G1	16.1 (8.6-45.7)	14.1 (6.6-45.7)
	G2	17.6 (7.6-38)	17.12 (8.7-19.5)

Data expressed as mean ± SD or median (min. - max.);

HEI: healthy eating index;

^a ^statistical significance after intervention (p < 0.05).

^b ^statistical significance between groups (p < 0.05).

Only participants in the G2 group experienced significant difference in percentage change in plasma γGT, TG, total-c and LDL-cholesterol, and β-carotene concentrations from baseline to post-intervention. From those variables G2 differed from G1 in a significantly greater improvements only in plasma γGT (-6.12% greater) and β-carotene concentrations (43.5% greater). The Lifestyle Changing Program with or without high dietary fiber intake had no significant effects on plasma glucose, HDL-c, creatinine, hsCRP, MDA and uric acid concentrations (Table 3).

**Table 3:** Blood markers of control (G1) and high dietary fiber (G2) groups at baseline (T0) and after 10 weeks of intervention (T1).

		T0	T1
Creatinine (mg/dL)	G1	1.4 ± 1.8	1.4 ± 1.1
	G2	0.83 ± 0.18	0.87 ± 0.15
γGT (U/L)	G1	28.4 ± 11.1	33.4 ± 25.2
	G2	32.3 ± 22.5	28.6 ± 19.5^a, b^
Glucose (mg/dL)	G1	99.0 ± 21.4	101 ± 18.3
	G2	96.9 ± 15.0	95.5 ± 15.5
Triglycerides (mg/dL)	G1	141 ± 73.4	150 ± 77.2
	G2	145 ± 57.2	129 ± 55.3^a^
Total Cholesterol (mg/dL)	G1	177 ± 41.2	181 ± 42.0
	G2	197 ± 39.0	182 ± 33.7^a^
LDL-c (mg/dL)	G1	104 ± 31.9	106 ± 34.4
	G2	124 ± 37.7	112 ± 30.5^a^
HDL-c (mg/dL)	G1	44.3 ± 9.8	45.0 ± 10.6
	G2	45.6 ± 11.8	44.2 ± 10.6
hsCRP (mg/dL)	G1	0.54 ± 0.51	0.51 ± 0.53
	G2	0.5 ± 0.4	0.40 ± 0.3
Uric acid (mg/dL)	G1	5.3 ± 1.6	5.6 ± 1.7
	G2	5.1 ± 1.7	5.0 ± 1.4
MDA (mmol/L)	G1	1.1 ± 0.2	1.0 ± 0.3
	G2	0.94 ± 0.28	0.95 ± 0.23
β-carotene (mmol/L)	G1	0.5 ± 0.2	0.5 ± 0.3
	G2	0.4 ± 0.1	0.6 ± 0.3^a, b^

Data expressed as mean ± SD; γGT: gamma-glutamyltranspeptidase; LDL-c: low density lipoprotein cholesterol; HDL-c: high density lipoprotein cholesterol; hsCRP: high-sensitive C-reactive protein; MDA: malonyldialdehyde;

^a ^statistical significance after intervention (p < 0.05).

^b ^statistical significance between groups (p < 0.05).

**Figure 1:**
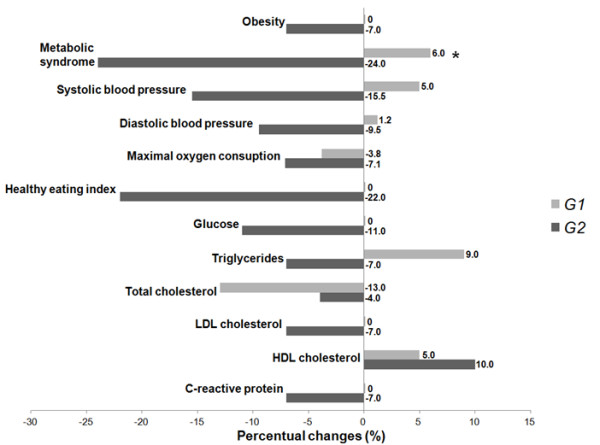
**Changes in altered clinical, dietetic and blood markers values after 10-week lifestyle changing program**. *p < 0.05.

The reduction in percent BMI change in the G2 group observed from baseline to post-intervention was associated with less incidence of obesity class III and class II. The 10-week high dietary fiber intake intervention combined with physical activity was associated with a reduction in percent change of MetS incidence from baseline to post-intervention, while no significant change was seen in the general educational group (Figure 1).

## Discussion

This study demonstrated improvements in multiple markers of HEI, body composition and metabolic indicators following a 10-week Lifestyle Changing Program aimed to assist overweight-obese subjects. The lifestyle intervention used in this study is an example of community education strategy, aimed to provide to all participants information and skills to assist them in achieving recommendations for diet and physical activity [36], additionally to participants in G2 it was offered practical strategies to assist in food choices. Because participants were left to choose which dietary behavioral changes they would incorporate into their lifestyles, it was anticipated that these behaviors would be easier to sustain long term. This builds upon well-established principles of health promotion, i.e. by making healthy choices will continue to make those choices [18].

There are clear limitations to the results presented in this study. The main limitation is that subjects on the high dietary fiber intake chose that intervention. Thus, the effects seen on the high dietary fiber intake may not be due entirely (or at all) to the LCP itself but rather to the fact that the subjects differed - they were more motivated and hence more likely to change their diet and lose weight. There are issues related to the method for collecting dietary data. Diet was assessed using a 24-hour dietary recall which has an important limitation for not capture intra-individual variability in food intake. Furthermore, overweight individuals are more likely to under-report their energy intake. Due to this underreporting, it is likely that the current estimates of calories could be underestimated and that dietary components played a larger role in this population's health.

Overweight-obese adults invited to participate in a 10-week high dietary fiber intake in combination with physical activity three times a week improved their HEI keeping stable their body composition, cardio-respiratory fitness and muscle strength. When LCP was associated with high dietary fiber intake there was an even better dietary quality along with reduced body sarcopenia and body adiposity. Furthermore, the high dietary fiber intake lead to a lower blood pressure and better plasma biochemical profile decreasing the incidence of MetS but without significance on markers of insulin resistance, inflammation and oxidative stress.

The adoption of general dietary guidelines, or *ad-libitum *intake, has been studied and shown promising results [37,38]. The free-choice approach to diet and physical activity has been described as a potential method to improve adherence to lifestyle changes [39]. Our dietary component allowed flexibility for individuals to modify their dietary intake, supported by offering practical strategies, such as suggestions, for ingredients substitution in recipes and different cooking methods.

This program may be more applicable to the community setting than individualized diet and exercise programs which require intensive input to facilitate behavioral change. Additionally the group-based approach may be less expensive than individualized strategies.

Few lifestyle intervention studies [18] have succeeded in counteracting obesity and MetS without controlling energy intake or physical activity, or aiming for large initial weight loss. Smaller behavioral changes, such as increasing dietary fiber intake, are an alternative strategy, and there is evidence that interventions without prescribed weight loss or energy intake targets can improve clinical markers.

The dietary components most strongly related to body composition improvement in the current study was the change in average of foods consumed. Although participants' HEI at baseline was classified as low, a significant greater increase occurred in G2 (20.3 points), compared to G1 (0.9 points). This is likely to reflect the improved dietary quality and better food choices consumed regularly during the intervention.

It is known that as dietary fiber intake increased, energy intake decreased and body weight and body fat decreased as well. Fiber adds to food weight and volume without increasing energy consumption. Thus, more food can be eaten without a commensurate increase in energy intake, or the same total volume of food can be consumed with loss total energy [40]. Total energy was not different between groups and moments. This is probably associated with underreporting of food consumption in obese individuals.

Diet and exercise interventions do not always improve plasma HDL concentrations, as observed by Janssen *et al*., [41] when plasma HDL concentrations did not improve after a 16-week dietary intervention, with and without aerobic or resistance exercise. Similarly, MetS participants in the Diabetes Prevention Program [42] experienced improvements in all MetS components except plasma HDL concentrations, indicating that plasma HDL concentrations are somewhat resistant to modification through general diet and physical activity changes.

There are few "free-choice" group-based lifestyle intervention studies demonstrating significant changes in BP. The observed reductions 15.5% in SBP and 9.5% in DBP exceeded that which might be expected to accompany weight loss in this trial, e.g. a 1 mmHg reductions in SBP for every 2 kg reductions in body weight [43], and are comparable to those seen after more intensive interventions [44,45].

The relationship between fiber intake and risk of cardiovascular disease has been noted in many studies [46-49]. Although the present study is quite small, the consistency of the findings about the benefits of dietary fiber intake is remarkable.

## Conclusion

Thus short-term LCP including high dietary fiber intake among obese subjects improved HEI and decreased body fat composition and plasma lipid concentrations leading to MetS incidence reduction.

## Competing interests

The authors declare that they have no competing interests.

## Authors' contributions

MSM performed the literature review, conducted the dietary interventions, analyzed body composition and wrote the manuscript. FM collected the blood analyzed the samples and supervised the data analysis. FHPB conducted the medical procedure, supervised the data analysis and reviewed the manuscript. RCD collected and supervised the physical activity and data analysis. KCPM analyzed the data and reviewed the manuscript. RCB was the mentor of the project, advice of the co-authors and principal investigator. All authors read and approved the final version of the manuscript.
